# Editorial: Women in Neonatology 2022

**DOI:** 10.3389/fped.2023.1288980

**Published:** 2023-10-10

**Authors:** Britt Nakstad, Ju-Lee Oei

**Affiliations:** ^1^ Department of Paediatrics and Adolescent Health, University of Botswana, Gaborone, Botswana; ^2^ Division of Paediatric and Adolescent Medicine, Institute of Clinical Medicine, University of Oslo, Oslo, Norway; ^3^ Department of Newborn Care, Royal Hospital for Women, Randwick, NSW, Australia; ^4^ Faculty of Medicine and Healthy, School of Paediatrics, University of New South Wales, Randwick, NSW, Australia

**Keywords:** neonatology, women, research, clinical, equity

**Editorial on the Research Topic** Women in Neonatology 2022

The Frontiers Research Topic of Women in Neonatology 2022 is an article collection in Frontiers in Pediatrics that aims to support gender equity, and hopefully raise awareness and promote the strengths of women in research, education, clinical care, and advocacy in the field of neonatal-perinatal medicine. Our community is richer and possibilities greater when we each actively advocate for and work towards equitable, diverse, and inclusive collaborations in the clinical setting and in research related to neonatology. We also sought to support and encourage the next generation of women and girls to pursue careers in research and to raise the experience necessary for future leadership. The purpose of this mission is driven by the recognition that gender equality in science is essential to ensure sustainable development as highlighted by UNESCO. Currently, less than 30% of scientists are women and to truly reduce this disparity, women must be encouraged to engage in STEM (Science, Technology, Engineering and Mathematics) careers (https://uis.unesco.org/en/topic/women-science).

In this collection of articles, the first or last author is a woman with expertise in neonatology. We received 11 submissions of which 4 were rejected. This collection includes 7 articles demonstrating the diversity of neonatology-related research led by women.

Chen and Law use visual attention analysis to study human factors in neonatal resuscitation preparations. They perform a 2-site randomized cross-over simulation study with eye-tracking glasses and randomize two methods to prepare epinephrine doses. Post-simulation, participants complete surveys and semi-structured interviews while viewing eye-tracked videos of their performance. Eye-tracked simulations provide human factors assessment of emergency neonatal code carts and epinephrine preparation and may help to choose the best technique.

Klerk et al. present a systematic review and meta-analysis on whether exposure to antibiotics is associated with a risk of necrotizing enterocolitis (NEC) in preterms. They search MEDLINE and EMBASE between 1990 and 2021 and find 370 papers acceptable for screening of outcomes. Their findings suggest an association between third-trimester maternal antibiotics and decreased risk of NEC. However, an increased risk of NEC was associated with prolonged use of empirical antibiotics that are known to reduce and unfavorably change microbiota diversity. The authors also find that prolonging empiric antibiotic treatment after birth could be associated with an increased risk of NEC. Based on these results, they advise using empiric antibiotics for as short a duration as possible and specifically, for newborn infants, to evaluate the need for guidelines beyond 36–48 h.

Kyokan et al. review evidence-based guidance for healthcare workers and stakeholders in choosing the most appropriate warming devices for clinically unstable neonates whose parents or family members cannot offer skin-to-skin care. The authors conduct a rapid review that includes a search for systematic reviews as well as randomized and quasi-randomized controlled trials that include searches for neonatal thermal care guidelines for the use of warming devices in low-resource settings. The search also includes requirements for resources and specifications of warming devices used in the nursery. The authors find no differences in the effectiveness of radiant warmers, incubators, and heated water-filled mattresses in maintaining body temperature but find that insensible water loss increases with radiant warmers. Seven guidelines covering the use of neonatal warming devices show no consensus about the choice of warming methods for clinically unstable neonates. They point out that radiant warmers allow fast access to the baby during the delivery period but in the unit, warming mattresses are more effective and economical as they are low-cost, effective, and low-electricity consumption devices that can be used for prolonged periods. For convalescing infants, incubators are best, especially for very premature infants where there is a prolonged risk of large insensible water loss for several weeks.

Mestan et al. review evidence to determine the relationship between placental dysfunction and placental injury impact on the fetus and newborn infant. This is a topic of growing interest in neonatal disease research. A search in Pubmed on the placenta and neonatal outcomes shows the publication of 5,741 papers and almost half during the last 7 years. The authors summarize the most common neonatal diagnoses that have been associated with placental dysfunction and discuss how recent technological advances may allow a shift to use valuable information from the placenta to guide neonatal management more effectively and to improve neonatal outcomes. The authors propose new ways of clinical management in which information from the placenta, with evaluation of the placenta histology as well through closely related biomarkers in maternal blood/urine, cord blood, and postnatal biospecimens, can be related to neonatal management. They conclude that opportunities to advance neonatal care using information related to the placenta with more rapid delivery of information to clinicians in NICU settings would lead to a rapidly evolving approach to bedside diagnosis and more timely management of multiple problems in the NICU.

Morkuniene et al. compare the applicability of regional and global standards to the Lithuanian newborn population by sex and gestational age, based on the prevalence of small or large for gestational age (SGA/LGA). They perform a retrospective analysis from 1995 to 2015 (618,235 newborns of 24–42 gestational weeks). They conclude that the regional population-based growth references represent the Lithuanian neonatal weight and length much more accurately than the global standard INTERGROWTH21st (IG-21, https://intergrowth21.tghn.org/), which provides the prevalence rates for SGA/LGA that differ from the true values.

Priyadarshi et al. review point-of-care bowel ultrasound appearances of the neonatal bowel in necrotizing enterocolitis (NEC) and provide several photos and figures to ease the reading and understanding. Taken together this is a useful tool for clinicians. The authors compare NEC findings to those seen in midgut-volvulus, obstructive intestinal conditions such as milk-curd obstruction, and slow gut motility in preterm infants on continuous positive airway pressure (CPAP) for CPAP belly syndrome. Priyadarshi et al. argue that bowel sonography is helpful when the diagnosis is unclear and NEC cannot be excluded. They propose that NEC is often over-diagnosed, due to a lack of useful biomarkers and the fact that the clinical presentation is similar to other neonatal conditions like sepsis. Thus, the assessment of the bowel using real-time ultrasound would allow clinicians to determine the timing of re-initiation of feeds and would also be reassuring based on specific typical bowel characteristics visualized on the ultrasound.

A second article from Priyadarshi et al. assesses, through prospective observations, bowel health in newborn infants between 27 and 43 weeks gestation. They compare conventional bowel sound auscultation using recordings with an electronic stethoscope, continuous 60-s recordings of bowel sound at a set region over the abdomen, to real-time bowel motility visualized by bowel ultrasound. Both methods are performed in 30 neonates on full enteral feeds without bowel pathology. A second investigator, blinded to the auscultation findings by the first investigator, conducts bowel ultrasound images using the same 12l Linear US probe. All recordings are analyzed for bowel peristalsis by the two methods. The authors find no correlation and different median time of peristalsis with ultrasound (58%), compared to acoustic assessment (88.3%, *p* < 0.002). Their findings call for better innovative methods to characterize bowel sounds in neonates, by integrating acoustic mapping with sonographic detection of bowel peristalsis, maybe using artificial intelligence.

This article collection is inspiring, informative, and substantially advances knowledge. It offers unique views of many aspects of neonatal care, theory, research, and methodology. Most importantly, it is a representation of the possibilities that women bring to the field of research and clinical experience in neonatology, one of the most research-active areas in pediatrics. This is a diverse and cross-cultural collection but may under-represent the potential of women in the global neonatal research community. We encourage women, especially those in lower-resourced settings and from countries where English is not the primary language, to actively advocate for and work towards equitable, diverse, and inclusive environments to advance clinical work and research in newborn medicine.

